# In Need of Renewal Rather Than Reconciliation: Why We Cannot Be Satisfied With Hospital Management’s Status Quo: Comment on "Dual Agency in Hospitals: What Strategies Do Managers and Physicians Apply to Reconcile Dilemmas Between Clinical and Economic Considerations?"

**DOI:** 10.34172/ijhpm.2022.6859

**Published:** 2022-03-13

**Authors:** Benjamin Ewert

**Affiliations:** Department of Health Sciences, Fulda University of Applied Sciences, Fulda, Germany.

**Keywords:** Hospital Management, Activity-Based Payment Systems, Professional Identities, Professional Hybrids, Hospital Reform

## Abstract

Activity-based payment systems enforce Israeli and German hospital professionals to continuously balance clinical and economic considerations. As argued this status quo is unsatisfactory due to two reasons. First, professional hybridity in hospital management is restricted to the physician versus manager dichotomy rather than a multifaceted-identity framework. Second, by depending mostly on serendipity rather than hospital professionals’ organizational leeway applied reconciliation strategies seem extremely temporarily and brittle. As concluded, alternative models of hospital funding and organization such as global budgets are urgently needed. In addition, hospital professionals have to be empowered to make effectively us from their hybrid identities.

 Modern hospitals are prototypes of complex healthcare arrangements. Above all, clinical and economic logics are often uneasily intermeshed in hospitals requiring sophisticated coping strategies from hospital professionals. In their recent article Waitzberg and colleagues sought to demystify this dilemma based on a qualitive study of activity-based payment systems in Israeli and German hospitals. Authors findings are of great value since they provide us to develop an empirically informed view of professionals’ ‘dual agency’^1^ by revealing that ‘physicians can be deeply involved with the hospital’s managerial aspects, and managers may aim at high quality of care.’^1^ Essentially, Waitzberg et al claim that existing tensions between clinical and economic rationales are not *per se* dilemmatic but could be reconciled if context factors are favorable and hospital professionals demonstrate extraordinary personal commitment to find a right balance.

 In this commentary, I will argue that Waitzberg and colleagues’ premises for succeeding hospital management are not entirely acceptable. Hence, I cautiously challenge authors’ conclusion that activity-based payment systems bear the potential to ‘create a win-win situation’^1^ (p. 10) allowing high-quality hospital care and efficiency gains. To elaborate my arguments, I will first refer to Waitzberg and colleagues’ understanding of dual agency that falls short to conceptual thinking of professional hybridity. Second, referring to authors’ empirical findings, I will argue that the alignment between economic and clinical considerations in Israeli and German hospitals depends too much on serendipity (ie, favorable context factors and professional engagement) and, therefore, needs to be supported by systemic renewal rather reconciliation.

 According to Waitzberg and colleagues’^1^ (p. 2) key premise hospital professionals are ‘“dual agents” when they are committed both to the patients and to the hospitals where they are employed.’ Hence, if coerced by systemic constraints clinicians and managers are capable to meet clinical and financial requirements. However, Waitzberg and colleagues’ notion of dual agency is similar but not synonymous with professional hybridity.^2^ Fueled by a binary perspective, dual agency implies that clinicians and managers, although ‘committed to more than one principal’^1^ (p. 9), are “normally” non-hybrids who act in an unambiguous and consistent manner. In contrast, literature suggests that professional hybrids “have some ‘unnaturalness’ as far as their being and identity are concerned”^2^ (p. 188). Rather than being physicians* or* managers, who unavoidably conduct tasks that go beyond their specialization, professional hybrids such as clinician-manager or manager-clinician are constituted by a complex combination of roles and values. Thus, while the enactment of dual agency seems basically a good starting point to cope with inevitable tensions in hospital management, fully acknowledging professional hybridity would better allow to move beyond ‘the dichotomy of two opposed professional groups’^1^ (p. 9).

 To theoretically capture professional hybridity in hospital management and beyond, applying a multi-faceted identity framework^3^ seems recommendable. The framework rejects dichotomous thinking by suggesting that professionals’ identity is composed by a *cross-cutting* identity, ie, the one of the professional, that is complemented by *nested* identities such as the ones of the practitioner or partner, street-level bureaucrat or citizen and manager or entrepreneur. Particularly, the hierarchical relation between cross-cutting and nested identities is crucial since it explains how professional hybridity, otherwise a rather chameleon term, is theoretically structured and practically unfolded. Consequently, manager-clinicians typically value managerial considerations higher than clinical ones as it is also stated in passing by Waitzberg et al^1^ (p. 9): ‘[H]ybrids typically adopt one role over the other.’ By contrast the manifestation of nested identities depends largely on individual dispositions and contingent context factors. A recent case in point are intensive care physicians who enacting their nested identity as citizens. Being politicized by the poor management of COVID-19 they pursue public interests (eg, restrictive lockdowns) to prevent hospitals from collapse. Likewise, Waitzberg and colleagues’^1^ conclusion that ‘economic and clinical considerations are less dichotomous than hitherto presented in the literature’ would have been even more compelling, if analyzed through a multi-faceted identity lens. In this regard, it would have been worth investigating whether managers’ capability to balance seemingly opposed principles is related to their *nested *identities: Are they enacting other identity facets like the one of the street level bureaucrat, citizen or entrepreneur while reconciling economic and clinical considerations? A multi-faceted identity framework would also enable us to investigate to what extent clinicians who are ‘deeply involved with the hospital’s managerial aspects’^1^ (p. 1) feel comfortable or uncomfortable in the face of their dual accountability. There are reasons to believe that a considerable number of medical professionals are skeptical towards managerial roles and duties.^4^ This does not mean that they have monolithic identities. Yet, doctors may have reason to value other nested identity facets than the one of the manager.

 As it should become clear by now, analyzing dilemmas caused by a ‘misalignment between economic and clinical consideration’^1^ (p. 10) through multi-faceted identity frames may lead to a different interpretation of professional hybridity. Problematically, the term dual agency restraints hospital professionals full range of motivations and priorities in the management of conflicting tasks. Recent research has demonstrated that ‘identity motives and identity work of (…) of doctors differ significantly’^4^ (p. 1477). Notwithstanding, authors’ empirical material showcases how professionals draw from multi-faceted identities *while *responding to dual agency schemes. For example, the reply by a Israelian physician (“…*But I also know that... there are loads of patients waiting [for a procedure]. So, the more I operate, the better... for the general public*,”^4^ (p. 6) indicates his/her nested identity as street-level bureaucrat who feels not only accountable for managerial or professional issues but also for the public interest. Moreover, empirical findings strongly hint to professionals’ dominating cross-cutting identity while “performing hybridity.” This becomes most apparent in the way German chief executive officers assess the supervision of coding “*When you shift a diagnosis further up or to the second or third position, then something shifts in the DRG [diagnosis-related group] reimbursement*”; “*there can be one [patient] who stays [hospitalized] for a long time and [another] one who stays a very short time*.* But the average tends to the optimal LoS [length of stay] for a particular procedure*”^4^ (p. 7). In these cases, clinical considerations are clearly subordinated to managerial ones. Conversely, physicians plan treatments ahead “*to prepare the patient and avoid having last-minute problems.*”^4^ For them, managerial concerns such as cost benefits are (at best) welcome windfalls. Thus, it is fair to say that Waitzberg et al present text-book examples of ‘incidental hybrids’^5^ (p. 412) that are ‘bound to the traditional professional mindset and values’^6^ (p. 7). Evidently, activity-based payment schemes impel hospital professionals to pursue fragile reconciliation strategies. Under this paradigm, the evolution of ‘willing hybrids’^5^ (p. 412) that would allow hospital professionals to actively reorganize their working environment seems rather unlikely.

 My second comment concerns the key learnings authors derive from their study. As it seems, an alignment of economic and clinical considerations in hospital management depends foremost on serendipity. Reaching at least a ‘fragile balance between high-quality care and financial sustainability’^1^ (p. 8) requires no less than a perfect fit between context factors and hospital professionals’ commitment to cope with economic constraints. This becomes most clear by comparing the three types of strategy. Findings suggest that Israeli and German hospital professionals fairly succeed in performing delicate tasks such as shortening patients’ length of stay, substituting materials and specialization despite context-specific incentive structures to do so. Having adapted themselves to economic and regulatory hospitals settings professionals increase efficiency with strenuous efforts. The same cannot be said with regard to the reshaping of hospital management. As reported, Israeli ‘physicians were not aware of the potential of coding to improve the billing of activities’^1^ (p. 7) while their German colleagues have obviously *learnt* to strategically gaming the DRG system,^7^ representing a most doubtful management skill. With regard to the third strategy, ie, reframing decision-making, success requires, both, ‘organizing professionalism’^2^ (p. 199) and ‘supportive organizational environments’^6^ (p. 2). Above all, modes of ‘[j]oint, multidisciplinary decision-making’^1^ (p. 8) need to be backed by professionals’ willingness to take collective action that is encouraged by hospital settings. Evidence to what extent the Israeli and German health systems differ with regard to this, indeed, very ‘strong reconciling strategy’^1^ would have been significantly complemented Waitzberg and colleagues’ study.

 To sum up, none of the identified strategies sufficiently relieve hospital professionals from the burden to cope with reconcile dilemmas. Authors display a rather grim reality where activity-based payment systems dictate professional agency within the limits of the clinician vs. manager dichotomy. Instead, moving ‘beyond activity-based funding’^8^ would allow professionals to enact their multi-faceted and hybrid identities. Alternative models to current hospital payment schemes provide a strong ‘focus on collaboration, regulated self-regulation and new tools to motivate professionals.’^8^ For example, a scheme from Central Denmark ‘separates the payment of hospitals from the management of performance’^9^ (p. 64) by operating with global budgets for individual hospital departments which then define their own performance goals. Thus, the utilization of global budgets emanates from hospital professionals’ collective expertise and department-wise prioritizations rather than centrally determined prospective payments. Primarily, global budgets provide physicians and managers with organizational leeway for self-governing their departments’ affairs (ie, management of cases and treatments) based on shared decisions. By productively interacting ‘with professional values and norms’^10^ (p. 362) global budgets seem more suitable to support the formation of willing hybrids.^6^ In sharp contrast, activity-based payment schemes, as illustrated by Waitzberg et al, provide a restrictive framework in which incidental hybrids need to cope with contingent context factors which are largely beyond their control. In conclusion, economic considerations prevail in hospital management over clinical ones as long as ‘efficiency savings and competition seems to be the only game in town’^11^ (p. 58). Against this backdrop, temporarily achieved alignments are unsatisfying compromises to the disadvantage of patients and care quality. In view of given clinicians’ disproportionate input of resources to accomplish brittle reconciliation strategies hospital management urgently needs reform. Needless to say, new institutional arrangements, ie, financing schemes and organizational models that empower rather than coerce hospital professionals, are indispensable. However, besides political support doctors and managers require also multi-professional training and education in order to jointly shape working environments in hospitals and rework management schemes in line with the global budget approach. Finally, hospital management renewal needs more efforts to effectively transform dual agents into professional hybrids that are being able to unfold their multi-faceted identities according to their own terms.

## Ethical issues

 Not applicable.

## Competing interests

 Author declares that he has no competing interests.

## Author’s contribution

 BE is the single author of the paper.
